# The *luxS *mutation causes loosely-bound biofilms in *Shewanella oneidensis*

**DOI:** 10.1186/1756-0500-4-180

**Published:** 2011-06-10

**Authors:** Agnes M Bodor, Lothar Jänsch, Josef Wissing, Irene Wagner-Döbler

**Affiliations:** 1 Helmholtz-Centre for Infection Research, Group Microbial Communication, Division of Microbial Pathogenesis, Inhoffenstr. 7, 38124 Braunschweig, Germany; 2 Helmholtz-Centre for Infection Research, Group Cellular Proteomics, Division of Microbial Pathogenesis, Inhoffenstr. 7, 38124 Braunschweig, Germany

**Keywords:** quorum sensing, autoinducer-2, *Shewanella oneidensis*, *luxS*, biofilm, secretome

## Abstract

**Background:**

The *luxS *gene in *Shewanella oneidensis *was shown to encode an autoinducer-2 (AI-2)-like molecule, the postulated universal bacterial signal, but the impaired biofilm growth of a *luxS *deficient mutant could not be restored by AI-2, indicating it might not have a signalling role in this organism.

**Findings:**

Here, we provide further evidence regarding the metabolic role of a *luxS *mutation in *S. oneidensis*. We constructed a *luxS *mutant and compared its phenotype to a wild type control with respect to its ability to remove AI-2 from the medium, expression of secreted proteins and biofilm formation. We show that *S. oneidensis *has a cell-dependent mechanism by which AI-2 is depleted from the medium by uptake or degradation at the end of the exponential growth phase. As AI-2 depletion is equally active in the *luxS *mutant and thus does not require AI-2 as an inducer, it appears to be an unspecific mechanism suggesting that AI-2 for *S. oneidensis *is a metabolite which is imported under nutrient limitation. Secreted proteins were studied by iTraq labelling and liquid chromatography mass spectrometry (LC-MS) detection. Differences between wild type and mutant were small. Proteins related to flagellar and twitching motility were slightly up-regulated in the *luxS *mutant, in accordance with its loose biofilm structure. An enzyme related to cysteine metabolism was also up-regulated, probably compensating for the lack of the LuxS enzyme. The *luxS *mutant developed an undifferentiated, loosely-connected biofilm which covered the glass surface more homogenously than the wild type control, which formed compact aggregates with large voids in between.

**Conclusions:**

The data confirm the role of the LuxS enzyme for biofilm growth in *S. oneidensis *and make it unlikely that AI-2 has a signalling role in this organism.

## Background

*Shewanella oneidensis *is a Gram-negative *Gammaproteobacterium *isolated from the sediment of fresh water habitats [1], and occasionally from water columns and clinical specimens [2]. The most investigated characteristic of *S. oneidensis *is its ability to use a broad spectrum of electron acceptors [3]. In our previous study, *Shewanella *species were shown to produce autoinducer-2, proposed to be a universal signal molecule in bacteria [4], and to contain its synthesis gene, *luxS *[5]. As *S. oneidensis *readily forms biofilms and lives in bacterial communities and since AI-2 is the product of the widely distributed *luxS *enzyme, AI-2 signalling would represent a possibility for this species to react to the bacterial density in its environment.

However it is under debate if the signalling role of AI-2 can be generalized. First, LuxS is not a designated enzyme producing AI-2 but rather an enzyme in the central S-adenosyl-methionine cycle [6]. Second, other bacterial species have no genes homologous to the AI-2 receptor of *Vibrio *species and seem just to transport AI-2 into the cells under nutrient limitation. There is an ABC-transporter in *Escherichia coli *and in *Salmonella typhimurium *which is specific for AI-2, but AI-2 taken up into the cell regulates only the expression of this transporter [7]. Recently, a *luxS *mutant of *S. oneidensis *strain MR-1 was shown to form a slightly reduced biofilm during the first 16 h of growth [8]. Since the wild type biofilm could not be restored by complementation with AI-2, the authors concluded that disruption of the activated methyl cycle rather than signalling by AI-2 caused the observed change in biofilm growth. This was supported by the observation of impaired growth of the *luxS *mutant on medium containing methionine, a component of the activated methyl cycle, as the sole sulfur source [8].

Here, we provide independent observations regarding the phenotype of a *luxS *mutant of *S. oneidensis *in comparison to a wild type control.

## Results and discussion

**Figure 1:**
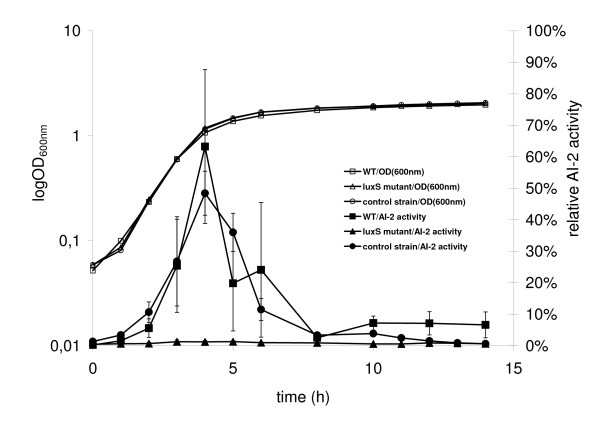
**AI-2 production of *S. oneidensis *wild type, *luxS *mutant and WT control strain**. All three strains were grown in LB at 30°C with shaking. Periodically, the growth was measured (OD_600 nm_) and sterile culture supernatants were collected and used to determine the produced AI-2. The AI-2 amount in the supernatant was detected by the *V*. *harveyi *bioassay and expressed in relative AI-2 amount. All strains grew at identical growth rates. The AI-2 production of the MR-1 wild type strain (WT) and the WT control strain (control strain) are indistinguishable, while that of the *luxS *mutant was at background level.

By targeted single-crossingover homologous recombination, the *luxS *mutant and the WT control carrying the same kanamycin cassette were constructed. The *luxS *mutant and the WT control grew at almost identical growth-rates in LB medium under aerobic conditions. The *luxS *mutant did not produce AI-2 (approx. 2% of positive control), while the WT control produced the same amount of AI-2 as the wild type. Furthermore, as observed previously with the wild type strain [5], the WT control accumulated AI-2 in the exponential phase in the medium and depleted it in the early stationary phase (Figure 1.).

### AI-2 removal from the culture supernatant

As AI-2 depletion from the medium is AI-2 dependent in *S*. *typhimurium *and in *E*. *coli *[7], we tested this possibility in *S*. *oneidensis*.

**Figure 2:**
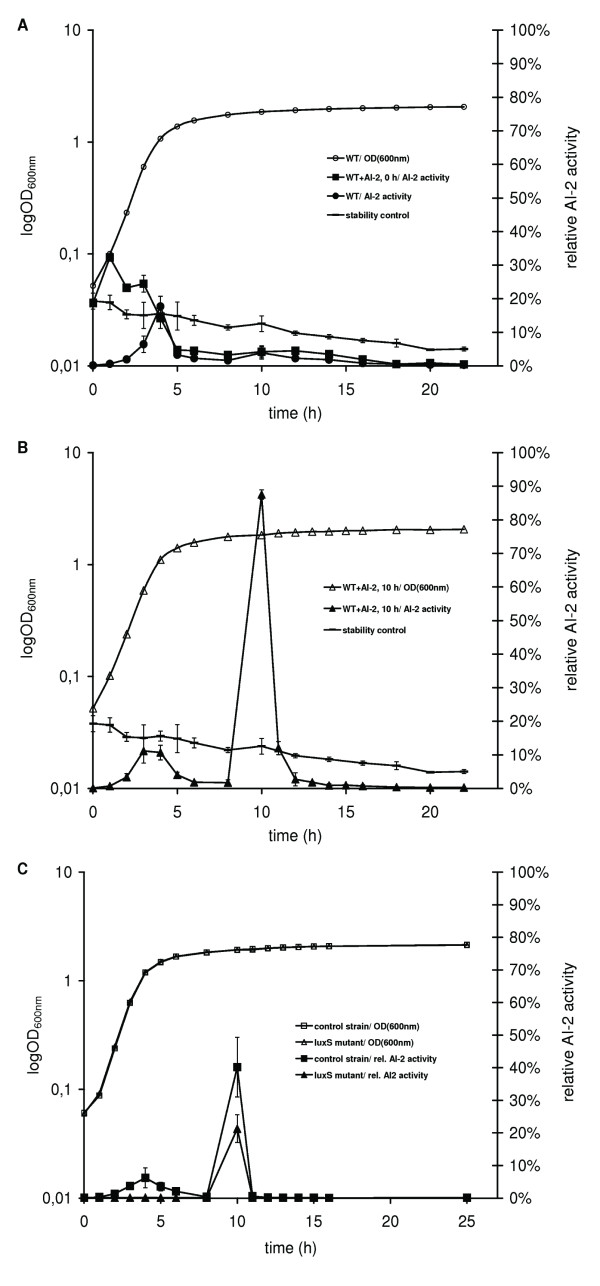
**AI-2 depletion by *S. oneidensis *wild type, WT control and *luxS *mutant**. (A) *S. oneidensis *depleted AI-2 added at the beginning of growth (WT+AI-2, 0 h) at the end of the logarithmic phase like the control culture (WT). (B) When the wild type strain was supplemented with AI-2 after 10 h of growth (WT+AI-2, 10 h), as indicated by the dashed line, AI-2 activity increased strongly. Still, the wild type depleted AI-2 within one hour. AI-2 had a low decomposition rate under the tested conditions (stability control). (C) When the *luxS *mutant and the WT control were supplemented with AI-2 after 10 h of growth, both strains depleted AI-2 within one hour.

As a first test, *S. oneidensis *wild-type was supplemented with AI-2 at two different time points: at the beginning of growth, to see if AI-2 depletion can be pre-induced by AI-2 before its natural onset, and after 10 h of growth, when AI-2 cannot be detected anymore in the medium, to determine if it is still depleted. As a result, AI-2 was depleted at the same time point in the native and supplemented cultures, indicating that AI-2 did not induce its depletion. Thus, its depletion appears to be growth-phase dependent (Figure 2A). When added after 10 h of growth, AI-2 disappeared within one hour, indicating that AI-2 depletion was still very active (Figure 2B).

To test if the *luxS *mutant lost this capability, synthetic AI-2 was added to both, the *luxS *mutant and WT control, after 10 h of growth and AI-2 levels were measured over time. Just like the wild type strain, both strains depleted AI-2 within 1 h after its addition, thus clearly indicating that AI-2 decrease is independent of the presence of AI-2 in this bacterium (Figure 2C).

By contrast, in *S*. *typhimurium *and *E*. *coli*, the expression of the ABC-transporter is AI-2 dependent and this transporter is specific for AI-2 [7]. Therefore, AI-2 uptake in *S. oneidensis *might be caused by a transporter with a broad substrate spectrum or a degradation mechanism which is expressed independently of AI-2 at the end of the exponential phase. This finding supports the hypothesis, that AI-2 for *S. oneidensis *is a metabolite which is imported under nutrient limitation.

**Figure 3:**
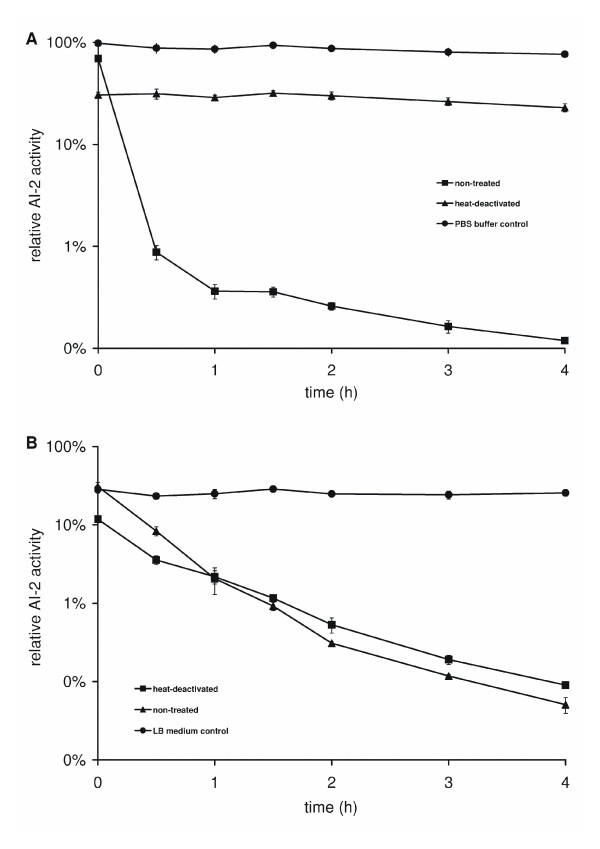
**Uptake and extracellular depletion of AI-2 by *S. oneidensis *wild type**. (A) Native cells of *S. oneidensis *washed in PBS depleted externally added AI-2 rapidly, while heat-killed cells were unable to do so. AI-2 was stable in PBS buffer. (B) AI-2 levels decreased in the non-treated as well as in the heat-inactivated supernatants. AI-2 was stable in LB medium.

To investigate if AI-2 is depleted by the cells or degraded extracellularly, the cells were pelleted and resuspended in PBS buffer after 10 h of growth, and then this cell suspension, its cell-free supernatant, and heat-inactivated control solutions prepared from these solutions were tested for AI-2 decrease. The living cells removed AI-2 from the medium within one hour, while the heat-killed cells left the AI-2 concentration unaffected, and thus AI-2 depletion is clearly cell-driven in *S. oneidensis*. Surprisingly, AI-2 decreased substantially also in the cell-free supernatants, although at a lower rate. There was no difference between heat-inactivated and untreated sterile supernatants. This decrease must therefore be caused by the reaction of AI-2 with heat-stable components of the cell-free supernatant, rather than by an enzyme (Figure 3A and 3B).

### Secretome analyses

The secretome of *S. oneidensis luxS *mutant and the WT control strain was compared in LB medium at the beginning of the stationary phase (OD_600 nm _= 1.5), shortly after the peak of AI-2 activity, when AI-2 would be expected to have induced extracellular proteins.

**Figure 4:**
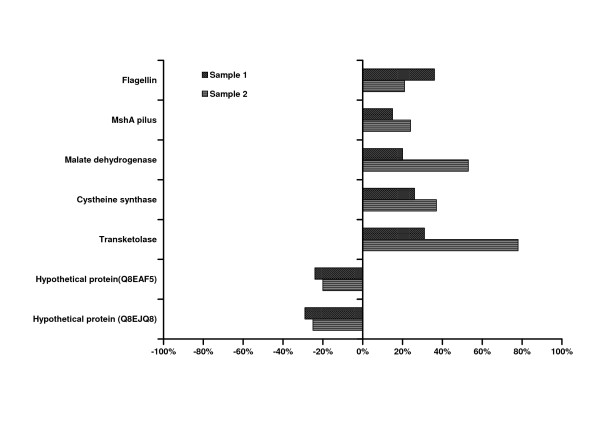
**Differentially regulated proteins in the *luxS *mutant**. Seven proteins were slightly but consistently differentially regulated in the *luxS *mutant compared to the WT control in the two tested samples. Two proteins related to motility - MshA pilin and flagellin - and three metabolic enzymes (malate dehydrogenase, cysteine synthase and transketolase) were induced, while two hypothetical proteins were repressed.

In two independent experiments, 172 proteins and 169 proteins, from which 103 were common in both experiments, were identified (see Additional file 1). Only eight proteins could be identified which showed differential regulation at moderately low cut-off limits, i.e. between 1.2 and 0.8 fold regulation (Figure 4). These cut-off limits are sufficient to detect regulation in view of the high sensitivity of the applied LC-MS method.

Two protein components related to motility, flagellin and MshA (mannose-sensitive hemaglutinin) pilin protein were slightly upregulated. Flagellin is a major component of the flagella (Interpro of EMBL-EBI). The MshA pilin constitutes the type IV pilus related to twitching motility [9].

In other *luxS *mutants, for example in *Campylobacter jejuni *[10] and in *Helicobacter pylori *[11], reduced motility has been observed. Both flagellar and twitching motility are involved in biofilm development in *S. oneidensis *and in other species. The *mshA *mutant of *S. oneidensis *could not adhere to the glass surface appropriately, while mutants without flagella or with paralysed flagella formed only non-differentiated, flat biofilms [9]. Similarly, flagellar motility in *E*. *coli *and twitching motility in *P*. *aeruginosa *was involved in spreading on a glass surface during biofilm formation [12,13]. Therefore upregulation of these proteins in the *S. oneidensis luxS *mutant would indicate a better glass coverage and eventually a different structure of the mature biofilm, which was actually observed in our biofilm experiment.

Three metabolic enzymes, namely cysteine synthase, malate dehydrogenase and transketolase, were slightly upregulated. These proteins might have been upregulated in response to the disrupted methyl cycle in the *luxS *mutant. They could either compensate for the lack of homocysteine or channel the accumulating S-ribosyl-homocysteine (SRH) into the carbon metabolism. Cysteine synthase conceivably compensates for the lack of homocysteine, since the product of the catalysed reaction, cysteine, is easily convertible to homocysteine. The connection of this enzyme to methylation processes is also supported by the fact that the gene for cysteine synthase forms one operon with the *luxS *gene in *Halobacillus halophilus *[14] and is located at a different position but followed by an O-methyl-transferase in *S. oneidensis*. The other two enzymes are involved in carbon metabolism. Malate dehydrogenase is an enzyme of the citric acid cycle. Transketolase provides a link between glycolysis and the pentose-phosphate pathway.

### Biofilm growth

**Figure 5:**
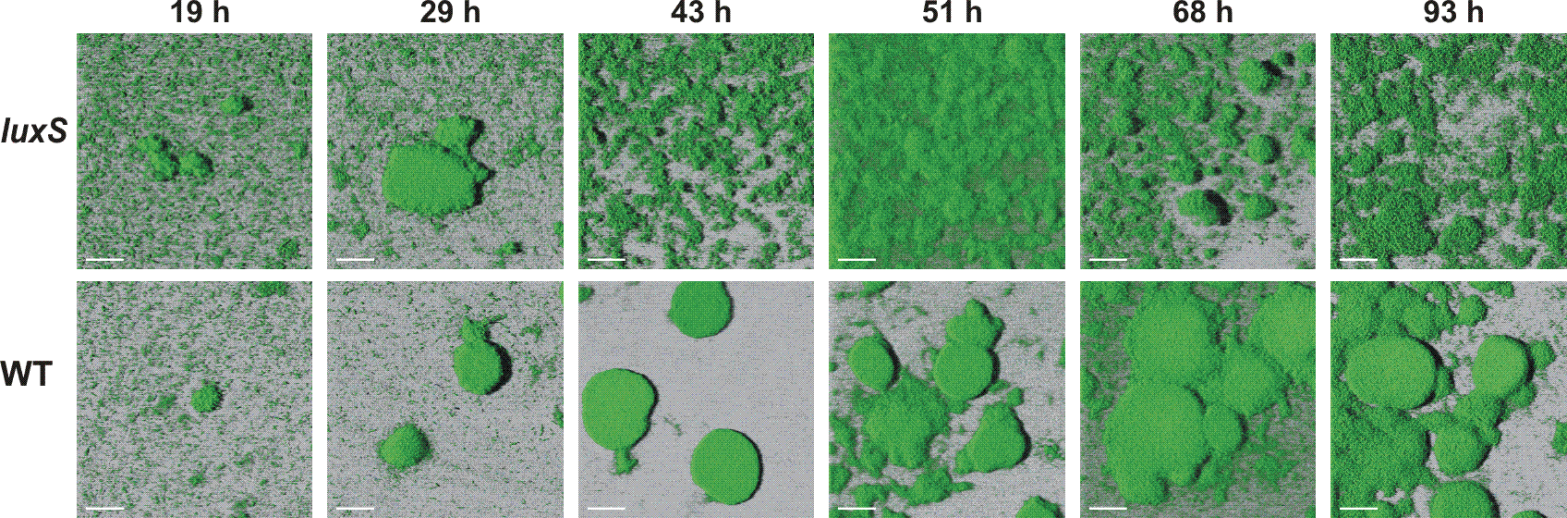
**Biofilm development of the *S. oneidensis luxS *mutant and the WT control strain**. Biofilms of both strains were grown on glass surfaces, which were placed in Petri dishes filled with SDM. At each time point, a glass slide was removed from the Petri dish and used for microscopic investigation. The pictures are selected from 2-5 records. Up to 9 h of growth, 2 D images from the fluorescence microscope were recorded with 40 × magnification, and these showed no difference between the two strains. After 19 h of growth, 3 D images could be recorded by Confocal Laser Scanning Microscopy. From 19 h until the end of the experiment, the *luxS *mutant formed a less-differentiated, loosely-bound biofilm, and the WT control tended to gather into tight clusters.

The *S. oneidensis luxS *mutant and the WT control strain were cultivated statically on glass slides in defined minimal medium. The biofilm of the *luxS *mutant showed a less-differentiated layer of loosely-bound cells which covered the surface evenly, while the WT control tended to form tight, flat and round-shaped clusters. In addition, the *luxS *mutant biofilm developed faster and after four days it was already in the detaching phase, while the WT biofilm was still developing (Figure 5. and Table 1.). At the early developmental stages, up to 9 h, the mutant and the control biofilms were indistinguishable: both strains displayed a homogenous single layer that gradually grew denser. On the second day, after 19 h and 29 h of growth, the mutant grew denser everywhere, while the wild type aggregated into clusters. This difference became most apparent on the second day, after 43 h, when the *luxS *mutant had a thicker biofilm forming a homogenous layer in contrast to the wild type clusters, as also reflected by the biofilm parameters. On the third day, after 51 h and 68 h of incubation, the mutant biofilm remained unchanged and appeared to have reached its maximum in volume and depth, while the control biofilm was still growing. On the fourth day, after 93 h, the mutant biofilm seemed to have arrived at the detachment phase due to its porous appearance and biofilm parameters, while the control biofilm was still developing.

**Table 1:** Biofilm parameters calculated by the PHLIP Matlab tool.

Time [h]	Total biovolume [μm3]		Substratum coverage [%]		Mean thickness [μm]	
	
	*luxS *mutant	WT control	*luxS *mutant	WT control	*luxS *mutant	WT control
19	9,3*10^5^	4,5*10^5^	15	6	15	6
29	2,9*10^6^ 2,8*10^6^	2,3*10^6^ 1,4*10^6^	15 20	9 7	16 17	16 15
43	7,2*10^6^ 8,3*10^6^	2,6*10^6^ 2,4*10^6^	32 65	21 18	15 14	9 8
51	4,2*10^6^ 3,2*10^6^	4,6*10^6^	51 30	35	25 19	11
68	7,9*10^6^	9,4*10^6^ 5,2*10^6^	57	50 43	22	12 8
93	3,9*10^6^ 4,0*10^6^	1,1*10^7^ 8,1*10^6^	39 43	54 81	14 11	13 17

Biofilm formation depends strongly on the medium used. In our hands, *S. oneidensis *was unable to produce substantial biofilms in complex media like LB, and also the frequently applied, complex, nutrient-limited biofilm medium LML [11,15] did not work well, but biofilm growth was observed in the defined *Shewanella *minimal medium (SDM). In the work of Learman et al. [8] a minimal medium different from SDM was applied for biofilm investigations. Under these conditions, the biofilm of the *luxS *mutant had 10% less biomass than the wild type after one day when grown in a microtitre plate under static conditions. After three days, the wild type biofilm had increased its biomass by 40%, while the mutant biofilm remained constant [8]. Under flow-through conditions, the *luxS *mutant initially showed a lower density of microcolonies (after 16 h), while no difference to the wild-type could be observed at a later stage of biofilm growth (48 h). These data cannot be compared to our results directly, because we used a different medium and a glass surface. However, the principle observations are similar: The dynamics of biofilm growth were different between wild-type and mutant, with the maximum of difference seen after 16 h in the flow through system of Learman et al. [8], and after 43 h in our experiment. Moreover, the mutant biofilm stopped growth earlier than the wild type in both studies, possibly indicating a problem with toxic intermediates. In contrast to Learman et al. [8] we observed a marked difference in the architecture of the mutant biofilm, which failed to develop the big clusters of the wild type.

The *luxS *mutation had different effects on biofilm formation in different species, but this may be the consequence of the cultivation conditions: flow-chamber vs. static conditions and type of medium used. In the flow chamber, the *luxS *mutant of *Klebsiella pneumoniae *formed a flat, more homogenous biofilm after 16-24 h of incubation in contrast to the mature, mushroom-like structures of the control [16]. The *luxS *mutant of *Aggregatibacter actinomycetemcomitans *developed a biofilm with reduced biomass and more sparse coverage after 60 h of incubation on saliva-coated coverglass in a flow-chamber [17]. Under static conditions, however, the contrary was observed. The *luxS *mutant of *Eikenella corrodens *formed a biofilm with higher biomass compared to the control on polystyrene surfaces of microtitre plates [18]. The *luxS *mutant biofilms of *Helicobacter pylori *exhibited the same structure, but contained more cells than the control [19]. And finally, the *luxS *mutant of *Lactobacillus reuteri *formed thicker biofilms than the control both *in vitro *and *in vivo *[20]. The only exception is the *luxS *mutant of *Salmonella typhimurium *that lost the ability to form biofilms on gallstones almost completely under static conditions and exhibited impaired biofilms on polystyrene pegs [21]. Remarkably, most *luxS *mutants investigated, except *S*. *typhimurium*, formed flatter biofilms in flow-chambers, while thicker biofilms developed in static systems compared to the wild type, and this latter observation is confirmed by the biofilm of our *luxS *mutant of *S. oneidensis*. Furthermore, all these *luxS *mutants tended to cover the surface more homogenously. Conceivably, the *luxS *mutants tends to form loosely-bound biofilms, but depending on the cultivation method they appear differently: in static systems, the cells are accumulating and form a thicker biofilm, while in a flow-chamber, the biofilms are flat, because the loosely-connected cells are washed away by the continuous flow.

## Conclusion

This study shows that the *luxS *mutation in *S. oneidensis *results in the development of loosely-bound biofilms. The concomitant slight upregulation of motility related proteins, which was detected in the secretome, may be one reason for this changed biofilm architecture, because it can lead to a weaker connection between the cells. Other changes in the secretome, e.g. upregulation of a homocysteine synthase, appear to be compensating for the metabolic defect caused by the lack of the LuxS enzyme. There is a very active cell-driven mechanism for AI-2 depletion in this bacterium, which is growth-phase dependent and not induced by AI-2, thereby indicating a role for AI-2 as a metabolite imported under conditions of nutrient limitation in *S*. *oneidensis*. Taking together the results of our study with those of Learman et al. [8], it seems unlikely that AI-2 has a signalling role in *S. oneidensis*.

## Methods

### Bacterial strains, plasmids and primers

Bacterial strains, plasmids and primers are listed in Tables 2 and 3.

**Table 2:** Strains and plasmids used in this study.

Strain	Description and genotype	Sources/References
***Shewanella oneidensis***		
MR-1	Wild type strain	BCC/[2]
*luxS*^-^_ins_	pKnock-Km plasmid inserted into the *luxS *gene	this study
WT_Km_	pKnock-Km plasmid inserted before the *luxS *gene	this study
***Escherichia coli***		
S17-1*λpir*	Biparental mating Tp^R ^Sm^R ^*rec*A *thi pro hsd*R RP4-2-Tc::Mu-Km::Tn7 λ*pir*	Biomedal (Spain)/[29]
pir-116	Maintaining of R6K ori plasmids F^- ^*mcr*A Δ(*mrr*-*hsd*RMS-*mcr*BC) Φ80d*lac*ZΔM15 Δ*lac*X74 *rec*A1 *end*A1 *ara*D139 Δ(*ara*, *leu*)7697 *gal*U *gal*K λ^- ^*rps*L *nup*G *pir*-116(DHFR)	Epicentre/[30]
DH5α	Subcloning routine F^- ^Φ80*lac*ZΔM15 Δ(*lac*ZYA-*arg*F)U169 *rec*Al *end*AI *hsd*R17(r_k_^-^, m_k_^+^) *pho*A *sup*E44 *thi*-1 *gyr*A96 *rel*AI λ^-^	Invitrogen/[31]
HB101	Carrier of the pRK2013 plasmid for triparental mating. F^- ^*sup*E44 *lac*Y1 *ara*-14 *gal*K2 *xyl*-5 *mtl*-1 *leu*B6 Δ(*mcr*C-*mrr*) *rec*A13 *rps*L20 *thi*-1 Δ(*gpt*-*pro*A)62 *hsd*SB20 λ^-^	[32]
***Vibrio harveyi***		
BB152, BAA-1119	*luxM*::Tn5; produces only AI-2	ATCC/[33]
MM77	*luxM*::Tn5, *luxS*::Cm^R^; produces neither AI-1 nor AI-2	ATTC/[34]
BB170, BAA-1117	*luxN*::Tn5; senses only AI-2	ATCC/[33]

**Plasmids**		

pKnock-Km	Km^R^; *ori*T; source plasmid for the suicide vectors into *Sh. oneidensis*	NCCB 3407/[24]
pEX18Ap	Amp^R^; source plasmid for a replicating plasmid into *Sh. oneidensis*	Max Schobert, TU Braunschweig/[35]

pPS858	Amp^R^, Gm^R^; source of the Gentamicin-GFP (Gm-GFP) cassette	Max Schobert, TU Braunschweig/[35]

pRK2013	Km^R^; helper plasmid for conjugation	DSMZ 5599/[36]

pKnock-*luxS*	Construction of *luxS*^-^_ins_ Km^R^; pKnock-Km containing an internal fragment of *luxS *of *Sh. oneidensis*	This study

pBA2106	Construction of WT_Km_ Km^R^, pKnock-Km containing an upstream fragment of *luxS *of *Sh. oneidensis*	This study

pEX18ApGm	For tagging *Sh. oneidensis *with gfp Km^R^, Gm^R^, the Gm-GFP cassette ligated into pEX18Ap	This study

**Table 3:** Primers used in this study.

Primer name	**Primer sequence**^a^	**Anneal. temp**.
CluxS_NotI_for	TGG CAG AGA ACT GTT TAG **gcggccgc **AAC AGG CTC GCT TGA CG	66°C
BluxS_BamHI_rev	ATG GCA TAG AGA TCT CCA **ggatcc **CAG GGC GAT ACA ACG CCA C	66°C
LuxS_EcoRI_beg	CAT TAC TTG **gaattc **TTA CCG TTG ACC ATA CTC	60°C
LuxS_KpnI_mid	CAT TGG A **ggtacc **TC AAT AAT TTC CAC ATC	60°C

^a^Nucleotides in bold indicate the built-in restriction sites.

### Culture media and growth conditions

Bacteria were cultivated aerobically in Erlenmeyer flasks by shaking at 160 rpm on a rotary shaker using standard microbiological techniques. Growth was monitored by measuring optical density at 600 nm (OD_600 nm_) with a Pharmacia Biotech spectrophotometer. *E. coli *strains were grown at 37°C in Luria-Bertani (LB) medium. *V. harveyi *strains were grown at 30°C in autoinducer bioassay (AB) medium [22,23]. *S. oneidensis *was grown at 30°C in LB. For biofilm growth, *S. oneidensis *strains were cultivated in *Shewanella *defined minimal (SDM) medium (31) with 20 mM lactic acid as carbon source. For counter selection of *E*. *coli *after conjugation, solid SM medium was used. *E. coli *strains were selected with ampicillin (Amp) at 150 μg/ml, kanamycin (Km) at 50 μg/ml and gentamicin (Gm) at 20 μg/ml. *S. oneidensis *was selected with Gm at 20 μg/ml, Km at 100 μg/ml after conjugation on plates and Km at 200 μg/ml in aerobic cultures, respectively.

### Experiments with addition of AI-2

In the first experiment with the wild type strain, four flasks were filled with the same volume of medium. Three of them were simultaneously inoculated with the overnight culture of the wild type. Two cultures were supplemented with AI-2 at a concentration of 5.3 μM at 0 h and 10 h of growth, respectively, while the third culture was used as the control for AI-2 production. The fourth flask contained sterile medium and was supplemented with AI-2 at 0 h of incubation to test AI-2 stability under the experimental conditions. From all cultures and the medium control, samples were taken periodically and tested for AI-2 levels. Experiments with the *luxS *mutant and the WT control were conducted in the same way except that AI-2 was added only after 10 h of growth.

To study AI-2 depletion in cells and culture supernatants separately, *S*. *oneidensis *wild type cells were harvested after 10 h of growth and suspended in PBS buffer. One half of the cell suspension was heated at 95°C for 20 minutes to kill the cells, while the other half remained native. The culture supernatant was filter-sterilized, and similarly to the cell suspension, one half of the supernatant was heat-inactivated, while the other half remained native. Control solutions were sterile PBS buffer and LB medium. After adjusting the temperature to 30°C, all solutions were supplemented with AI-2 at a concentration of 5.3 μM. Then samples were taken periodically and tested for AI-2 levels.

### Construction of the *luxS *mutant and WT control in *S. oneidensis*

The *luxS *mutant and the WT control were constructed via single homologous recombination between a suicide plasmid and the chromosome of the wild type strain. The suicide plasmids were derived from the conditionally replicating plasmid pKnock-Km [24] by directional PCR cloning. For the *luxS *mutant, a coding fragment of the *luxS *gene, approximately 0.2 kbp long, was amplified with the primers LuxS_*Eco*RI_beg and the LuxS_*Kpn*I_mid and then ligated into the *Eco*RI and *Kpn*I restriction sites of the pKnock-Km plasmid. The resulting plasmid was first maintained in *E. coli *pir-116, then transformed in *E*. *coli *S17-1λpir and finally introduced into *S. oneidensis *wild type strain by biparental mating. The mating mixture was plated on SDM medium, as this medium lacks essential amino acids for *E*. *coli *to grow. To ensure that pure *S*. *oneidensis *colonies were obtained, the clones were streaked on LB agar with ampicillin at 150 μg/ml and kanamycin, and single colonies were selected and tested for mutation by PCR and sequencing (data not shown). For the WT control, an approximately 0.6 kbp long fragment upstream of the *luxS *gene with 17 bp spacing was amplified with the CluxS_*Not*I_for and BluxS_*Bam*HI_rev primers and then ligated into the *Not*I and *Bam*HI restriction sites of the pKnock-Km plasmid. The resulting plasmid was introduced into *S. oneidensis *wild type as described previously. The WT control was verified by PCR.

### Conjugation

Conjugation proved to be the only method to introduce DNA into *S. oneidensis*. Both biparental and triparental mating were applied, but the latter method proved to be more efficient. For biparental mating, *E. coli *S17-1 λ*pir *with the appropriate plasmid (donor strain), and *S. oneidensis *wild type (recipient strain) were grown to OD_600 nm _= 1, which corresponds to about 10^8 ^cells/ml. 1.5 ml of each culture was harvested by centrifugation at 4.000 × g for 3 min at room temperature (RT) and washed twice in 1 ml LB. After one more centrifugation, the pellets were dissolved in 30 μl LB. Controls of both cultures separately and conjugation mixes were prepared and spotted onto mating disks, which are sterile mixed cellulose ester membranes (Millipore, MF type without triton) placed on LB agar plates. After incubation for one day at RT, the cells were dissolved in 1 ml PBS buffer at RT, and 200-300 μl of the suspension was plated onto SM agar with Km (100 μg/ml). Appropriate orange clones appeared after 1-2 weeks at RT. For triparental mating, the donor strain was an *E*. *coli *strain, which optimally maintained the mobilizable plasmid, the conjugating strain was *E*. *coli *HB101 with the helper plasmid pRK2013, and the recipient strains was *S. oneidensis*. These strains were prepared for conjugation and the clones were selected as described for biparental mating.

### GFP-tagging of the *luxS *mutant and the WT control

To enable microscopic documentation of living bacteria, the *luxS *mutant and the WT control were labelled with the green fluorescent protein (GFP). For this purpose, the Gentamycin-GFP (Gm-GFP) cassette of the pPS858 plasmid was inserted into the *Bam*HI site of the pEX18Ap plasmid containing an origin of transfer, which enabled conjugation into *S*. *oneidensis*, and *Col*E1 *ori*, which enabled plasmid replication in *S*. *oneidensis*. The resulting pEX18ApGm plasmid was conjugated by triparental mating into the *luxS *mutant and the WT control, respectively, and selected as described above.

### Detection of AI-2

AI-2 was determined in the culture supernatant using the *V. harveyi *bioassay with the sensor strain *V. harveyi *BB170 as published previously [23]. The sensor strain *V*. *harveyi *BB170 was cultivated to bright light intensity with a final OD_600 _of 1.0-1.1. This culture was diluted 1:5000 in AB medium, resulting in the working solution of the sensor strain. 180 μl of this working solution were added to 20 μl of the test samples, reference media and controls pipetted on white microtitre plates (NUNC, Roskilde, Denmark). Four replicates were measured per test sample and six replicates for the control. The microtitre plates were incubated at 30°C with agitation at 650 rpm. Luminescence was measured hourly in a Victor Wallac Luminescence Reader (Perkin Elmer) for 6 h. Sterile culture supernatants from *V*. *harveyi *BB152 and MM77 served as positive and negative controls, respectively. Chemically synthesized AI-2 [25-27] diluted in AB medium was used as an additional positive control at a concentration of 5.3 μM. The AI-2 amount present in the sample was expressed in relative AI-2 activity. First, the fold change of induction of luminescence was calculated by dividing the luminescence of the samples by the luminescence of the sterile reference medium. The maximum of fold induction determined during 6 h was referred to as AI-2 activity. The relative AI-2 activity was calculated by dividing the AI-2 activity of the sample by that of the positive control.

### Biofilm cultivation

Biofilms of the GFP-tagged *S. oneidensis luxS *mutant and WT control were grown in a static system in defined *Shewanella *minimal medium (SDM) with 20 mM lactic acid as carbon source [1]. First, the strains were pre-cultivated at 30°C in LB medium for 4 h to reach an OD_600 nm _of 0.7-1. Then, 10 biofilm dishes were prepared. For each of them, 12 ml of the LB culture were pipetted on a microscopic glass slide placed in a Petri dish, and after 45 min of incubation at 30°C, each microscopic slide was transferred to another Petri dish with 12 ml of fresh SDM medium. These biofilm dishes were also incubated at 30°C. At each time point, one biofilm dish was sacrificed for microscopy: the microscopic glass slide was washed in sterile PBS buffer and covered with a glass slip. The biofilm structures on the microscopic glass slides were examined with a fluorescence and a confocal laser scanning microscope. The fluorescence microscope was an Olympus BX60 microscope equipped with Plan objectives (Olympus, 40 x, 0.65; 10 x, 0.25) and with a fluorescence lamp (Olympus, model U-ULS100Hg), for which the light source was provided by a 100 W high pressure mercury burner (Olympus, model BH2-RFL-T3). A Colorview digital camera and cellA imaging software were used for documentation. The confocal laser scanning microscope (CLSM) was a Fluoview 1000 (Olympus) microscope equipped with UPlanSApo objectives and simultaneously detected bright field and EGFP images (20 mW 561 nm solid state laser and 30 mW 488 nm multiline argon laser with 2% power respectively). The monochrome series of images of the CLSM was measured along the optical axis with 1 μm increments. The Imaris X64 5.7.2 software was used for creating the 3 D images. The biofilm parameters like biomass, substrate coverage and mean thickness, were calculated by the Phlip Matlab toolbox [28].

### Secretome analysis

The *S. oneidensis luxS *mutant and the WT control strain were cultivated at 30°C in LB for 5-5.5 h until an OD_600 nm _of 1.5 was reached. Then, the whole 100 ml culture was centrifuged at 4.000 × g for 6 minutes at 4°C and the culture supernatant was sterilized by filtration (0,22 μm). For secretome analysis, proteins were precipitated by sodium deoxylate (DOC). Peptides were generated by trypsine digestion. The peptides were labelled by different iTRAQ reagents (Applied Biosystems) according to the manufacturer's instructions.

iTraq labelled peptides were first separated using strong cation exchange on a Mono S PC 1.5/5 column with an gradient over 35 minutes from 100 buffer A to 35% buffer B (A = 25% acetonitrile, 0.1% formic acid; B = 25% acetonitrile, 0.1% formic acid, 500 mM KCl) at a flow rate of 150 μl/min. Fractions of 150 μl were collected, dried in a SpeedVac centrifuge and purified using ZipTipμ RP 18 material. The separation of the peptide samples was performed using a bioinert Ultimate nano-HPLC system (Dionex). 10 μl of each sample (containing up to 1 μg peptides) were injected, and peptides were purified and concentrated on a C_18_-PepMap pre-column (0.3 mm i.d. × 5 mm, 100 Å pore size, 3 μm particle size) at a flow rate of 30 μl/min in 0.1% TFA. Subsequently, peptides were separated on an analytical 75 μm i.d. × 150 mm C_18_-PepMap column (Dionex, 100 Å pore size, 3 μm particle size) at a flow rate of 200 nl/min. The gradient (Solution A: 0.1% formic acid, 5% acetonitrile; solution B: 0.1% formic acid, 80% acetonitrile) started at 5% and ended at 60% B after 120 minutes. MS and MS/MS data were acquired using a Q-TOFmicro mass spectrometer (Waters, Milford Massachusetts; USA). Doubly and triply charged peptide ions were automatically selected by the MassLynx software (MassLynxx 4.1 1 b) and fragmented for a maximum of 18 seconds per peptide. MS data were automatically processed and peak lists for subsequent protein identification by database searches were generated by the MassLynx software. Database searches were carried out with an in house MASCOT server using the EMBL *Shewanella oneidensis *database. Proteins were only accepted as identified when at least one unique peptide showed an individual score above 20, which indicated identity or extensive homology (p < 0.05) using the given settings (Enzyme: Trypsin; Max. missed cleavages: 1; Fixed modification: iTRAQ (K); iTRAQ (N-term); Oxidation (M); Peptide tolerance: 0.4 Da; MS/MS tolerance 0.3 Da).

## Competing interests

The authors declare that they have no competing interests.

## Authors' contributions

AGB and IWD planned the investigations and wrote the manuscript. AGB performed the experiments. LOJ and JOW measured the secretome samples. All authors read and approved the final manuscript.

## Supplementary Material

Additional file 1 **Table S1**. All proteins identified in the first and the second experiment are listed (prot_extracted). The two lists were compared and common proteins were selected (common_prot). Then the proteins were sorted according to their regulation and regulated proteins were selected (regulated_prot). Click here for file
